# Use of Spectacles

**Published:** 1874-06

**Authors:** 


### USE OF SPECTACLES.

When you find that you are beginning instinctively to go to the window or the open door when you take up a paper or book to read, it is time for you to purchase a pair of spectacles; then get those of the lowest magnifying power, for if they magnify too much the eyes will get prematurely old—will fail rapidly. If, while reading, you find an inclination to stop and wink the eyes, as if to clear them, you need spectacles, or if you have them already you require older ones. A good way to rest the eyes from reading or fine sewing is either to close them for a few minutes, or look at something a long distance off; this gives great relief.

Do not purchase glasses on the street, or at cheap places, for there is great danger that the glasses are not alike—have not exactly the same focus, or are made of soft glass, which is easily scratched and then older ones are soon required. The value of Brazilian pebbles, which is a natural glass, is their greater hardness; hence, not easily injured, last a great while and the eyes get old very slowly. But if the glasses are soft, are mismated, having unequal focal points, the eyes are strained and get old in a short time, especially if both foci are not in the centre of the glass. In selecting glasses choose those which enable you to see well without a strain, for habitual straining of the eye ages it rapidly; at the same time avoid getting those of a stronger power than is needed.

Glasses should be washed with cold or warm water at least every morning, and should be wiped several times during the day with fine buckskin, and nothing else; paper scratches them, and so does flannel, and a handkerchief is seldom clean enough for the purpose.

As the eye is the most delicate organ of the body it should be treated with great care, and economy in spectacles is a great, a life long misfortune. The eyes should not face a light; it is better to have the book in such a position as to have the light come on the page over the shoulder.

In reading by artificial light the eyelids should be shaded in such a way that the full glare does not fall on them, but on the paper or book, and cease using them as soon as they begin to feel tired.

